# Particleboards with Recycled Material from Hemp-Based Panels

**DOI:** 10.3390/ma17010139

**Published:** 2023-12-27

**Authors:** Electra Papadopoulou, Iouliana Chrysafi, Konstantina Karidi, Andromachi Mitani, Dimitrios N. Bikiaris

**Affiliations:** 1 CHIMAR HELLAS S.A., 15 Km National Road, Thessaloniki—Polygyros, 570 01 Thermi, Greece; k.karydi@ari.gr; 2 Laboratory of Advanced Materials and Devices, Department of Physics, Faculty of Sciences, Aristotle University of Thessaloniki, 541 24 Thessaloniki, Greece; iochrysa@physics.auth.gr; 3 Department of Forestry, Wood Sciences and Design, University of Thessaly, V. Griva nr.11, 431 00 Karditsa, Greece; amitani@uth.gr; 4 Laboratory of Polymer and Colors Chemistry and Technology, Department of Chemistry, Faculty of Sciences, Aristotle University of Thessaloniki, 541 24 Thessaloniki, Greece; dbic@chem.auth.gr

**Keywords:** hemp shives, wood chips, recycling, particleboards, soy flour, circular economy, thermal degradation

## Abstract

This research addresses the current need for sustainable solutions in the construction and furniture industries, with a focus on environmentally friendly particleboard. Particleboards were made from a mixture of virgin wood chips and hemp shives, which were then mechanically recycled and used to make new lightweight particleboards. Phenol–formaldehyde resin with 25% *w*/*w* phenol replacement by soybean flour (PFS) was used as the binder for the lignocellulosic materials. Laboratory analyses determined the resin properties, and FTIR confirmed the structure of the experimental PFS resin. The thermal properties of all the resins were evaluated using thermogravimetric analysis (TGA). The panels were manufactured using industrial simulation and tested for mechanical and physical properties in accordance with European standards. The FTIR study confirmed good adhesion, and the TGA showed improved thermal stability for the recycled biomass panels compared to virgin biomass panels. The study concludes that lightweight particleboards can be successfully produced from recycled hemp shive-based panels, providing a sustainable alternative to traditional materials in the construction industry.

## 1. Introduction

For approximately 10,000 years, humanity has relied on wood, incorporating it into virtually every facet of daily existence, a practice that continues to this very moment. Nevertheless, with the escalating felling of trees, an increasing amount of carbon dioxide remains trapped in the atmosphere, giving rise to adverse consequences for our planet, including a heightened global temperature and increasingly acidic oceans. This prompts us to inquire about potential alternatives to wood chips for manufacturing the same array of products [1,2,3].

Industrial hemp shives are a promising candidate. They surpass wood chip products in terms of being lighter, more robust, and cost-effective. An acre of hemp yields as much cellulose fiber as four acres of trees. Furthermore, hemp matures to a usable fiber stage in just 100 days, while trees require a significantly longer timeframe, spanning anywhere from 50 to 100 years, to reach a comparable stage [4].

Hemp is growing in popularity as a building product. For years, it has been used to make rope, insulation, composites [5,6], bioplastics [7,8,9], and other industrial materials. In the area of wood chip-based panels, scientists continue to investigate its uses with undiminished interest.

Auriga et al. (2022) [10] studied seven variants of particleboards with a density of 650 kg/m^3^ with 10–25% hemp shive substitution in different layers. For their production, a typical urea–formaldehyde resin was used as a binder. It was found that the hemp shive-based panels had internal bond (IB) values similar to particleboards made entirely of industrial wood chips and slightly higher modulus of rupture (MOR) and modulus of elasticity (MOE) performance, while the ones with 25% hemp shives exhibited a reduction in swelling values.

In a similar work, Rimkiene et al. (2023) [11] made particleboards by mixing fibrous hemp shives and corn starch at the levels of 5, 10, 15, and 20%. Before use, corn starch was mixed with sodium metasilicate. Additionally, expandable graphite ES 350 F5 was used as a flame retardant. The Tubiquard 44 N non-ionic fluorocarbon resin in the form of water dispersion was used as a hydrophobizer for the water- and oil-repellent finish. During this research, the influence of the composition of the mixture, the processing of raw materials, and technological parameters on the operational properties of the board were evaluated. It was found that a starch content of 15% and water content of 10% produced panels with the best properties. Additional processing of hemp shives can increase bending strength by more than 40%. If the pressing is increased from 5 t/m^2^ to 15 t/m^2^, the density of the boards increases by about 1.5 times, and the bending strength is more than 50%. The additives used made it possible to reduce the water absorption of the boards up to 16 times and obtain non-flammable boards.

Alao et al. (2020) [12] developed hemp shive (*Cannabis sativa* L.)-based particleboards of low density (477–581 kg/m^3^) using as bonding materials typical urea–formaldehyde resin, formaldehyde-free acrylic resin (Acrodur^®^), and bio-based soy resin (Soyad™). Hemp shive boards based on soy resin showed the best results in tensile and bending strengths, 0.43 and 13.9 MPa, respectively, while panels with UF resin had the best thickness swelling performance.

Hemp shives were also used by Zvirgzds et al. (2022) [13] for the production of lightweight particleboards (300 ± 30 kg/m^3^). The cold pressing method was used to produce hemp shive boards with Kleiberit urea formaldehyde resin as a binder. Additional components, such as color pigments and wood finishes, were added to test improved features over raw board samples. The water absorption test confirmed that the chosen type of binder decays swiftly in water, and hemp shives soak up a lot of water. Thickness swelling was 20% lower for boards with a larger shive group and a further 35% lower with additive–water base coating. The hemp shive-based panels had significantly lower bending strength values than particleboard from wood chips.

Fehrmann et al. (2023) [14] studied the use of hemp shives in particleboards of very low densities (213–309 kg/m^3^) and three types of adhesives, namely bio-epoxy (EPX), phenol resorcinol formaldehyde (PRF), and emulsifiable methylene diphenyl diisocyanate (MDI). Before use, the hemp shives were milled and fractionated into fine (F), medium (M), and coarse (C) particles. It was found that panels with MDI gave the best overall performance.

In all of the above studies, the particleboards were made from virgin hemp shives and wood chips and a variety of bio-based or petrochemical resins, which raises concerns due to the chemicals involved. Additionally, the use of virgin biomass in a product such as particleboard, which consumes large amounts of biomass, carries the risk of intensive land use and a potential contribution to deforestation. Moreover, the production of virgin biomass for particleboards requires resources such as water, fertilizer, and energy, and the scale of these inputs can potentially contribute to environmental degradation and increase the environmental footprint of the production process [15].

One way to reduce the use of virgin biomass resources while managing panel waste is to reuse this waste in the production of particleboard as an alternative raw material to virgin biomass. The recycling of particleboard with wood chips is a topic that has been studied by various scientists. New recycled wood-based panels have been produced on a laboratory scale, and their properties have been studied [16,17,18,19]. However, no literature references were found on the recycling of panels based on hemp shives, such as those produced in the present study. In this way, this project is new and innovative.

The proposed recycled hemp shive-based panels contribute to the conservation of natural resources, allow more land to be used for food production, help to manage waste panels by effectively diverting them from landfills, and promote a more sustainable approach to their production. This work highlights the contribution of hemp shive-based panels to the circular economy. Moreover, their production using bio-based resin provides consumers with products that are healthier and more environmentally friendly than panels made with petrochemical-based adhesives.

**Figure 1:**
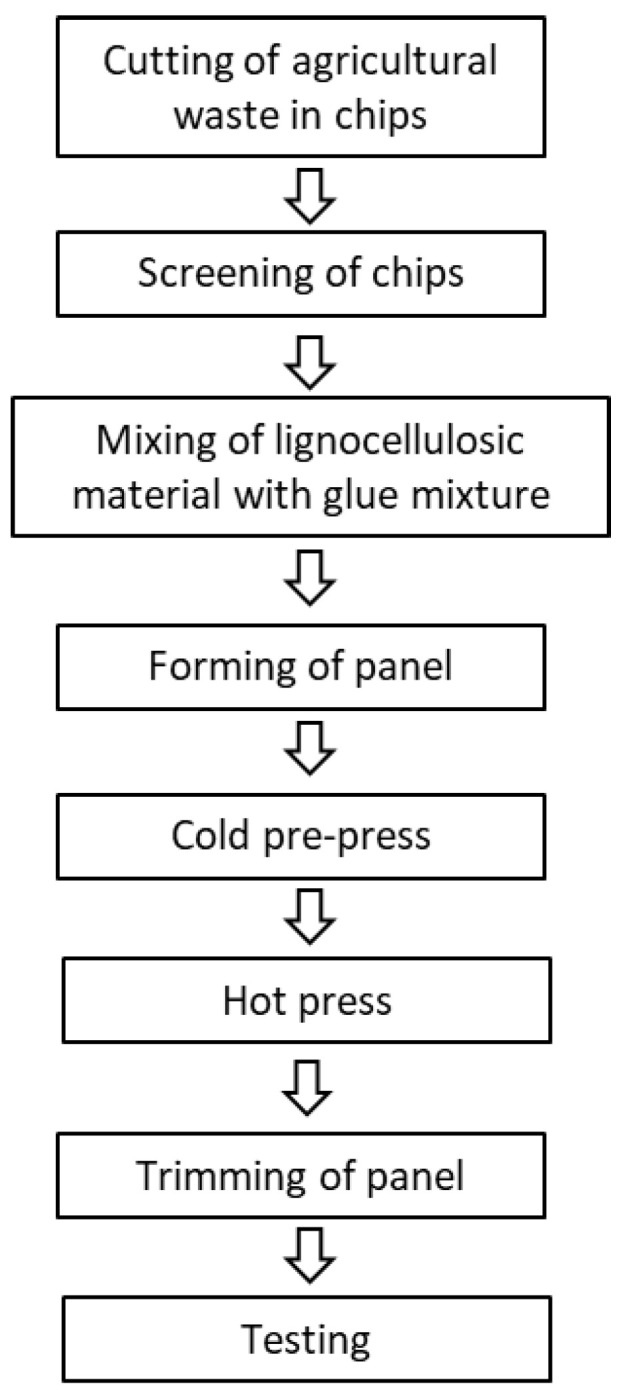
Steps of particleboard production [20].

In our study, lightweight particleboards were produced by replacing virgin wood chips or hemp shives with recycled biomass resulting from the mechanical destruction of particleboards made from virgin wood/hemp materials. The boards were produced according to the typical industrial practice, which includes the following steps (Figure 1):

In order for particleboards to be of guaranteed quality, they must meet certain specifications depending on their final use. In Europe, industrial standards are used to define the specifications of commercial particleboard for various applications, as well as the lower acceptable price limits and the way in which the evaluation tests are carried out (ΕΝ 312:2010). As far as the classification of particleboards in terms of their formaldehyde content is concerned, this is based on the standard ΕΝ13986:2001.

For the manufacturing of these panels, we developed a protein-based phenol–formaldehyde resin (PFS) to increase the bio-content of the final product. Typical phenol–formaldehyde (PF) and urea-formaldehyde resins (UF) were used as reference.

Typical PF resins are produced in three steps [21,22,23,24,25,26]. First, formaldehyde is added to phenol using an alkaline catalyst (often NaOH) (step one). The temperature is then raised to boiling temperature and the hydroxymethylphenols polycondensate to form a mixture of polymer chains with different molecular weights. This is the second step, and the product at this stage is called “Resitol”. The third step is the final cross-linking and hardening of the polymer, which takes place during the production of the wood chip-based panels under high pressure and temperature.

In the case of urea–formaldehyde (UF) resins, their typical synthesis takes place in two basic stages: (a) methylation, where addition reactions of formaldehyde (F) to urea (U) take place at neutral to alkaline pH, and (b) polycondensation, which takes place at slightly acidic pH. At this stage, high molecular weight polymer chains are formed, linked together by ether bonds and methylene bridges [27,28,29].

The chemical and thermal properties of all resins and panels were evaluated with typical laboratory analysis, Fourier transform infrared spectroscopy (FTIR) and thermogravimetric analysis (TGA).

No literature references were found for the recycling of hemp shive-based panels and their reuse in the production of new particleboard. This work is, therefore, considered innovative.

## 2. Material and Methods

### 2.1. Lignocellulosic Materials and Chemicals Used

In the present study, hemp shives were supplied by the Greek company KANNABIO (Volos, Greece), while wood chips were supplied by the Greek particleboard factory AKRITAS (Alexandroupolis, Greece). The hemp shives were 1.5–3 cm long and 0.2–0.5 cm wide; their density was 90–91 kg/m^3^. The chips were 1.3–2.5 cm long and 0.2–0.5 cm wide, with a density of 110–112 kg/m^3^.

For the synthesis of resins, the chemicals used were industrial-grade aqueous solutions of phenol (44.2% *w*/*w*) and formaldehyde (47.6% *w*/*w*). Technical grade urea (100%), formic acid (10% *w*/*w*), and sodium hydroxide (NaOH-50% *w*/*w*) were purchased from Elton Group Chemicals (Attica, Greece), while defatted soy flour (Prolia FLR 200/90) with a protein content of 50% *w*/*w* was given by the Cargill Company (Amsterdam, The Netherlands).

### 2.2. Synthesis of Phenolic Type Resins

The typical process was followed for their synthesis, as described in the literature [21,22,23,24,25,26]. Specifically, phenol, sodium hydroxide, and water were added to a glass reactor equipped with a stirrer and a thermometer, and the mixture was cooled to 35–40 °C. Formol was gradually introduced while maintaining the temperature below 50 °C. The reaction mixture was stirred at 55–65 °C for 1.5 h, followed by raising the temperature to a boil and subjecting the mixture to reflux for 1 h. The progress of polycondensation was assessed through viscosity changes, and once it reached the desired level, the resin was subsequently cooled to room temperature. In the case of PFS resin, soy flour was added together with phenol. The ratio of the resins’ raw materials is shown in Table 1.

Tests were also carried out with higher percentages of phenol replacement, but the resulting preparations were not homogeneous, and they were considered unsuccessful. For this reason, resins with up to 25% *w*/*w* phenol replacement were used.

### 2.3. Synthesis of Urea–Formaldehyde Resins

The formation of urea-formaldehyde (UF) resins through the reaction of urea and formaldehyde is essentially a two-step process, typically involving an initial alkaline methylolation followed by a subsequent acidic condensation [27,28,29]. The synthesis of UF resins in this study followed the typical route. The needed urea was split into two parts. Initially, Urea-I was reacted with formaldehyde at an alkaline pH (7–8)—using sodium hydroxide as a buffer—at temperatures between 90 and 95 °C for 2 h (methylolation step). Acid was then added to lower the pH to 5.0–5.3 to allow the polymerization phase to begin. Frequent viscosity measurements were taken with a Brookfield viscometer, and when the target viscosity was reached, the pH was increased to stop the polymers from increasing in size. The second amount of urea (Urea–II) was then added, and the resin was allowed to react for a further 24 h at a temperature of 25–30 °C before use. The ratio of the resin’s raw materials is shown in Table 2.

### 2.4. Determination of Resin Properties

Quality control of all resins was carried out by determining their physico-chemical properties using standard laboratory analytical methods. Typical properties include determination of dry matter (solids), pH, viscosity, free formaldehyde, buffer capacity (only for UF resin), water tolerance, gel time, alkali content (only for PF type resins), and specific gravity. In particular:

Dry matter was measured according to the guidelines of the ASTM D4426-01 (2006) standard. The solid content was calculated as a percentage, determined by the final mass-to-initial mass ratio.

pH was determined by direct measurements at 25 °C using a CRISON pHmeter (Crison, Barcelona, Spain) device.

Viscosity was measured at 25 °C by using a Brookfield viscometer (DVEELVTJ0 Digital (AMETEK Brookfield, Middleboro, MA, USA) and it was expressed in cP.

Free formaldehyde content was measured according to the standard ISO 11402:2004. Buffer capacity was measured by titration with 0.1 N H_2_SO_4_.

The tolerance to water was determined by the amount of water that can be added to a solution of 5 g of resin until the solution becomes cloudy.

The gel time of the resins was assessed at 100 °C. A test tube containing 5 g of the prepared resin was placed in boiling water, and consistent stirring was maintained throughout the test. The gel time for the sample was determined as the elapsed time until further stirring was no longer possible.

Alkali content was determined by dissolving the resin in distilled water and titrating it to pH 3.5 with 0.1 NH_4_CI using a pH meter.

Specific gravity was measured at 20 °C using a hydrometer.

The thermal degradation of the resins was assessed with thermogravimetric analysis (TGA) using a Labsys Evo 1100 instrument (Setaram Instrumentation, Lyon, France). The uncured resins were heated from room temperature to 600 °C with a constant flow of air and nitrogen gas set at 50 mL/min at a heating rate of 20 °C/min.

FTIR spectra of the samples were acquired using a Cary 670 spectroscope manufactured by Agilent Technologies (Palo Alto, CA, USA), equipped with a diamond attenuated total reflectance (ATR) accessory (GladiATR, Pike Technologies, Madison, WI, USA). Infrared absorption spectra were collected in the range of 4000 to 450 cm^−1^, with a resolution of 4 cm^−1^ and 32 co-added scans. A baseline correction was applied to the obtained spectra, and they were further normalized for analysis. The analysis of the resins was performed in their liquid form before curing.

### 2.5. Production of Particleboards

Particleboard is composed of lignocellulosic elements bonded together with an adhesive under heat and pressure. For the evaluation of hemp shive biomass (virgin and recycled), particleboards were produced using the newly developed PFS resin as an experimental binder and typical UF and PF resins as reference binders.

Hemp shives and wood chips were first oven-dried to a moisture content of 3–6%. After drying, the material was sent to the gluing machine to be mixed with the glue mixture consisting of the liquid resins and water. Ammonium chloride was also used as a hardener for the UF resin, while no hardener was used for the PF and PFS resins. The ratio of the glue mixture components in each case was resin/water = 12/1 for PF and PFS resins and resin/water/hardener = 3/1/0.2 for UF resin. Inside the gluing machine, the glue mixture was sprayed in the form of very small droplets. The adhesive level was 9–11% (dry adhesive to dry biomass). The adhesive-impregnated biomass pieces were mechanically laid out to form a board (the aim is to produce an even mat). The mat was then cold pressed, followed by hot pressing (15–35 kg/cm^2^ for particle boards with a density of 0.4–0.8 g /cm^3^) at temperatures of 150–200 °C. The pressing time is proportional to the thickness of the particleboard and is approximately 0.3–0.4 min per millimeter of finished product thickness. The panels were then trimmed and smoothed to obtain a smooth and flat surface and, finally, cut to the appropriate dimensions to obtain the necessary samples for the evaluation of their properties.

Lightweight particleboards were produced with a density of 500 kg/m^3^, both with virgin wood chips and hemp shives, which were used as reference boards, and with recycled board chips by mixing them with wood chips and hemp shives in different proportions. In all cases, the particleboards were produced according to the above-mentioned procedure and production parameters. In total, 5 types of panels were prepared, as shown in Table 3.

### 2.6. Characterisation of Particleboards

The properties of all the panels produced in this study were measured according to the European standards listed in Table 4 and Table 5, and the results classify the panels into some of the categories listed in these tables.

Although it is not possible to make an absolute identification of the above categories of industrial production of wood chip-based panels and petrochemical adhesives with the laboratory panels produced in this work, the above information gives us a “guide” to the target values of the particleboard properties of the project.

Thermogravimetric analysis (TGA) was also used to study the thermal degradation of the particleboards. A small piece of each board weighing 4 ± 0.5 mg was heated from room temperature to 900 °C under a nitrogen atmosphere at the rate of 20 °C/min. An empty alumina crucible was used as a reference. Fourier transform infrared spectroscopy (FTIR) was also employed.

## 3. Results–Discussion

### 3.1. General Properties of Resins

The properties of the experimental and typical resins that were synthesized and used in this work for particleboard preparation are shown in Table 6.

As can be seen, the properties of the reference resins UF and PF are within the accepted limits of the industry and close to the properties reported in other scientific works [30,31,32]. The experimental PFS resin has properties close to the typical PF resin and is, therefore, acceptable for use. However, it should be noted that although it has a higher viscosity, it has a slower gel time and a higher free formaldehyde content. Typically, the higher the viscosity of a polymer, the higher the degree of branching, and, therefore, fewer sites are available for further cross-linking when measuring gel time and less free formaldehyde is available in the polymer. In this case, the higher viscosity is probably not due to a denser branching of the polymer chains but because the soy itself is a polymer, and this contributes to the overall viscosity of the polymer [33,34,35,36,37].

### 3.2. Fourier Transform Infrared Spectroscopy of the Resins and the Particleboards

**Figure 2:**
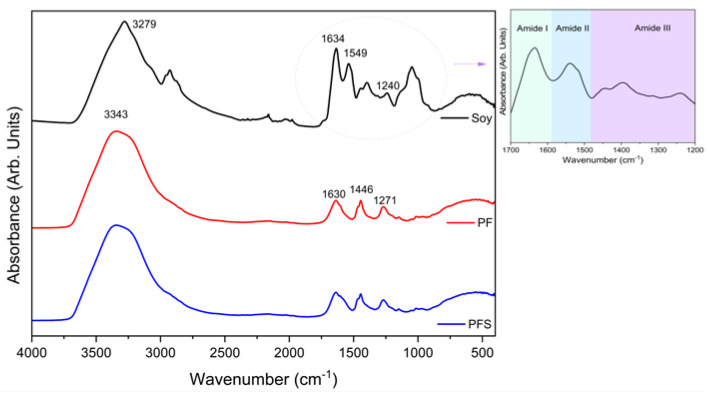
FTIR spectra of soy flour, PF, and PFS.

FTIR analysis was employed to evaluate the chemical structure of the new PFS resin. For comparison reasons, the spectra of PF resin and soy flour are depicted in Figure 2. PFS and PF resins were in liquid form, while soy flour was a powder. From the soy flour spectrum, a broad, sharp peak at 3279 cm^−1^ can be distinguished, which is due to the free and bound N–H and O–H groups [38]. The peaks at 1634, 1549, and 1240 cm^−1^ are due to the C=O stretching vibration of the amide I bond, the N–N bending vibration of the amide II bond, and the C–N stretching and N–H bending vibration of the amide III bond, respectively [38,39].

Regarding the spectrum of phenol–formaldehyde, a broad peak at 3343 cm^−1^, which is due to the stretching vibrations of the -OH groups in phenol, can be seen. The absorption peaks at 1630 cm^−1^ and at 1446 cm^−1^ are characteristic of formaldehyde and are due to the stretching vibrations of the C=C bonds. The peak at 1271 cm^−1^ is due to stretching vibrations of the C–O–C ether linkages. A weak peak located at 1150 cm^−1^ is attributed to stretching vibrations of the C–C bonds of phenol [40].

Concerning the phenol/formaldehyde/soy flour composite resin, no deviations from the PF spectrum are observed. The main peaks corresponding to soy are in the same area as those of phenol–formaldehyde and, due to its smaller percentage, cannot be distinguished in the spectrum.

**Figure 3:**
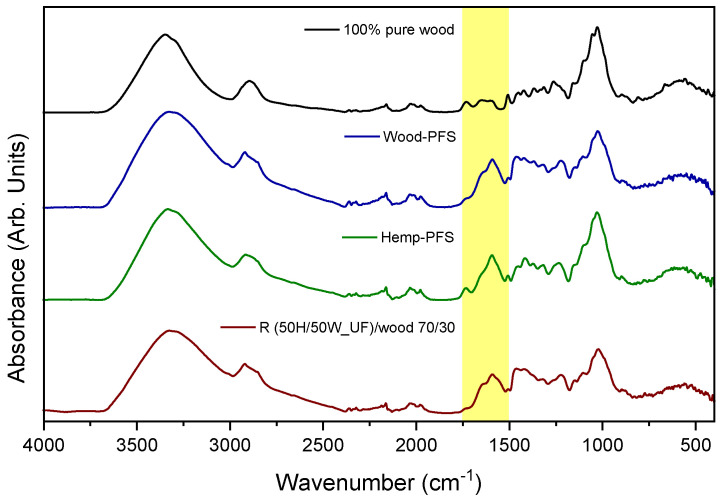
FTIR spectra of wood as a reference and the particleboard wood chips–PFS, hemp shives–PFS, and the recycled R (50H/50W_UF)/wood chips 70/30.

The analysis of the chemical structure of the particleboards was also carried out by means of infrared spectroscopy. First, the spectrum of a piece of wood chip without the presence of resins was taken for comparison, while the spectra of particleboards with the presence of resins were then presented. As shown in Figure 3, the particle board spectra are similar to that of pure wood, as the percentage of resin is quite small compared to wood chips/hemp shives and is characteristic of lignocellulosic compounds. The peaks between 2500 and 4000 cm^−1^ are due to the stretching vibrations of the O–H hydroxyl bonds of cellulose and the stretching vibrations of the C–H bonds of cellulose and hemicellulose. The peak at about 1050 cm^−1^ is due to the stretching vibration of C–O bonds of hemicellulose and cellulose [41,42,43]. Comparing the spectra of the boards with that of pure wood, a peak between 1500 and 1750 cm^−1^ can be distinguished; this is attributed to the existence of resins and specifically due to amides II. A decrease in the intensity of the peak at 1050 cm^−1^ is also observed on the particleboards. This is probably affected by the amide groups of the resins, suggesting a modification in the hemicellulose and cellulose bonding environment, leading to changes in the intensity of specific vibrational modes associated with C–O bonds.

### 3.3. Thermogravimetric Analysis of the Resins and the Particleboards

**Figure 4:**
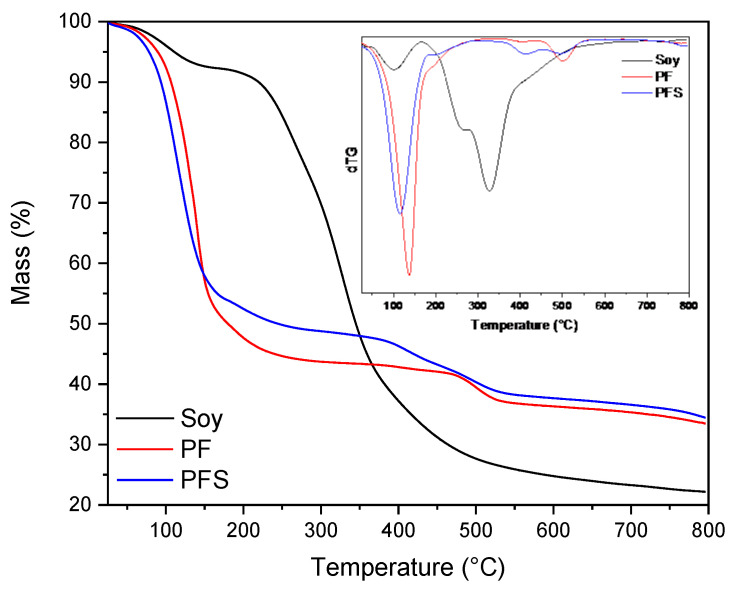
TGA thermogram of mass loss and dTG versus temperature curves of soy, PF, and PFS.

A thermogravimetric analysis (TGA) of the resins was also performed. Figure 4 shows the thermogram of soy flour, PF, and PFS resins. Concerning soy flour, it is clearly distinguishable from both the mass loss and the dTG curve as a function of temperature that degradation occurs in two stages. In the first stage, there is a mass loss of approximately 8% up to 168 °C, attributed to water evaporation and the degradation of the quaternary structure of the protein, with the maximum decomposition rate occurring at 100 °C. Beyond 100 °C, the protein denatures its subunits and promotes the formation of protein aggregates through electrostatic, hydrophobic, and disulfide bond exchange mechanisms [44]. The second stage is attributed to the degradation of the peptide bonds in the soy protein backbone. Simultaneously, various gases such as CO, CO_2_, NH_3_, and H_2_S are produced [42]. This stage ranges from 168 °C to 800 °C, with the maximum degradation rate at 327 °C, resulting in a mass loss of about 70%. Finally, approximately 22% of residual carbon mass can be distinguished.

Moving to the thermogram of PF, as shown, the resin’s degradation occurs in two stages. In the first stage, which extends up to 337 °C, water evaporation up to 100 °C and the decomposition of formaldehyde that remained unbounded in the resin take place [45,46]. The maximum degradation rate is found at 137 °C, with 67% of the mass degraded. The second stage extends up to 800 °C, resulting in a mass loss of about 10% and a maximum degradation rate at 500 °C, likely due to the cleavage of methylene bonds and the main ring structure being destroyed [45]. Finally, there is a remaining mass of approximately 33% that has not been degraded.

Regarding the thermogram of PFS, it can be observed that the composite resin exhibits a similar shape to that of pure PF. In the derivative of the mass loss diagram, there is an evident enlargement of the peak around 410 °C, and the second decomposition stage starts at lower temperatures, around 290 °C. In comparison to the pure resin, PFS shows a slightly higher percentage of remaining mass (35%), with the maximum degradation rate occurring at approximately 113 °C. Moreover, it can be observed that the main degradation stages take place at slightly lower temperatures than PF. This could be attributed to the interaction between the complex system of soy and the phenol–formaldehyde.

**Figure 5:**
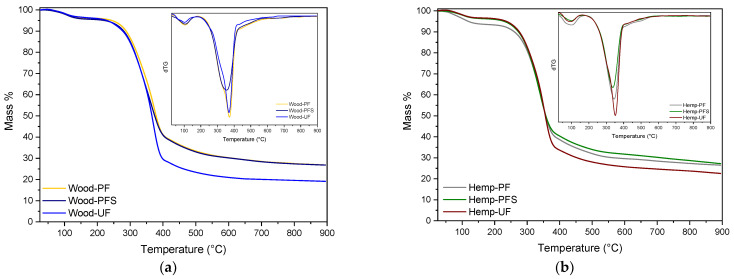
TGA thermogram of mass loss versus temperature curves of the wood chip (**a**) and hemp shive (**b**) particle boards prepared with PF, PFS, and UF.

The thermal performance of panels was also studied with thermogravimetric analysis (TGA). Initially, thermogravimetric analysis was carried out on the reference samples with 100% wood chips and hemp shives using the three different thermosetting resins: UF, PF, and PFS (Figure 5). As can be seen in the mass loss versus temperature thermogram, the shape of the curves is influenced by the fact that both hemp shives and wood chips are based on lignocellulosic compounds [7]. As shown in Figure 5a, two main degradation steps are evident, with the first extending up to about 180 °C, primarily due to the removal of moisture, and the second extending up to 750 °C, with a maximum degradation rate occurring at 367 °C for wood–PF and wood–UF and at 354 °C for wood–PFS. In the case of the hemp shive boards, the first degradation step occurs until approximately 175 °C, while the second persists until 610 °C. The maximum degradation rate for hemp shives–UF is at 350 °C, for hemp shives–PF at 345 °C, and for hemp shives–PFS at 338 °C. During the second stage, the degradation of wood and hemp, specifically hemicellulose, followed by cellulose and lignin, takes place [7]. Simultaneously, the resins decompose. Additionally, a percentage of residual mass ranging between 19% and 30% is observed, resulting from the formation of biochar from the wood and hemp, as well as from a quantity of resin that has not fully degraded, as confirmed by the resin thermograms. When comparing the particleboards with different resins, better thermal stability is observed in wood chip boards prepared with PF. In the case of hemp shive boards, those with PF and PFS exhibit slightly better thermal stability compared with that of UF, which is attributed to the stronger cross-linkages and inherent chemical structures of PF and PFS resins.

Next, the particleboards containing partly recycled materials were examined. A common behavior was observed, similar to that in the initial boards. The excess mass falls within the range of 26% to 30%. The first stage of degradation occurs between 45 °C and 176 °C, while the second stage extends to approximately 650 °C. The maximum degradation rate occurs during the second stage and is found to be between 335 °C and 357 °C.

**Figure 6:**
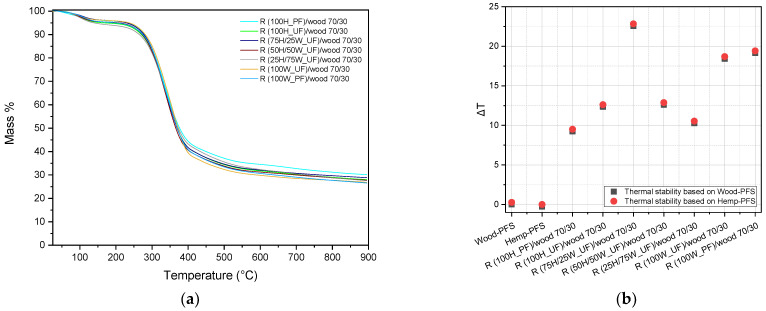
TGA thermogram of the recycled particleboards (**a**) and the comparison diagram of the thermal stability of the particleboards (**b**).

For a more comprehensive study of the recycled particleboards, a comparison of their thermal stability was conducted, with the initial particleboards consisting of 100% wood chips and 100% hemp shives, using the PFS resin, as studied in Figure 5. The thermal stability was assessed based on a 2% mass loss and is presented in Figure 6b. The black square points refer to the temperature difference between the wood–PFS (reference, T_2%_ = 81.7 °C) and the other particle boards, while in the red circles, the hemp–PFS board is used as a reference temperature (T_2%_ = 81.5 °C). It is first observed that both the hemp shive and wood chip particleboards exhibit similar values, as the mass loss in the two non-recycled boards is approximately the same; thus, the points are almost the same. Furthermore, the thermal stability of all the recycled particleboards is higher than that of the original ones, with the sample denoted as R (75H/25W_UF)/wood 70/30 being the most thermally stable. The increase in thermal stability can be attributed to the higher resin content in the recycled particleboards. The degradation of the cured resins initiates at approximately 200 °C, rendering the boards more thermally stable when compared to pure hemp shives or wood chips. During the recycling process, the resin may undergo further curing or cross-linking, which enhances its heat resistance and overall stability. Recycling also results in a reduction in moisture content within the particleboards. Lower moisture levels lead to improved thermal stability, as moisture can compromise the structural integrity of the boards and reduce their resistance to heat.

### 3.4. Mechanical Behavior of Particleboards

**Figure 7:**
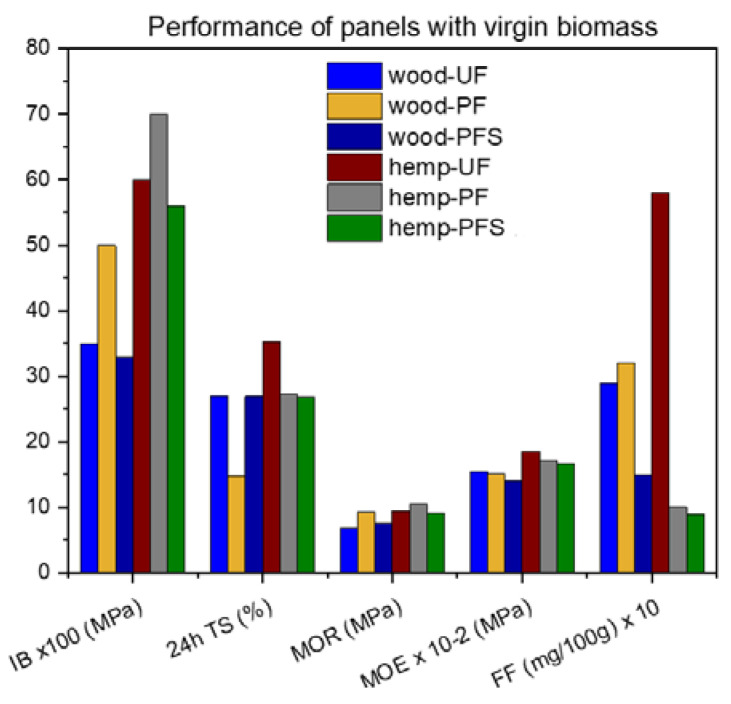
Performance of particleboards with virgin biomass and reference and experimental resins.

All resins synthesized had appropriate properties and were used for the formulations of particleboards. The test results (averages) for prepared particleboards are shown in Figure 4, Figure 5, Figure 6 and Figure 7, and their discussion follows.

The performance of panels made from virgin biomass and reference resins is illustrated in Figure 7. The results show that hemp shive-based panels perform better in terms of mechanical properties (IB, MOR, MOE) regardless of the resin used. However, these panels perform slightly worse in terms of thickness swelling. This finding is consistent with the results of other studies [10] and is to be expected, as the cellulose that gives strength to composite materials such as particle board is found in higher concentrations in the hemp feedstock (72%) than in wood (42%) [47]. It is also known that hemp can rapidly absorb large amounts of water (up to 3 times its own weight) during material preparation [48,49].

Comparing the different resins on the same biomass, in the case of wood chips, the panel made with PF resin has a much higher water tolerance than the panels made with UF and PFS resins. This observation does not apply to hemp shive-based panels. Regarding the formaldehyde content of the panels, the hemp shive-based panels with PF or PFS resins have the lowest content, while the UF resin has extremely high formaldehyde content. This is because the chemical bond between phenol and formaldehyde is stronger than that between urea and formaldehyde. The results are consistent with other studies [50].

**Figure 8:**
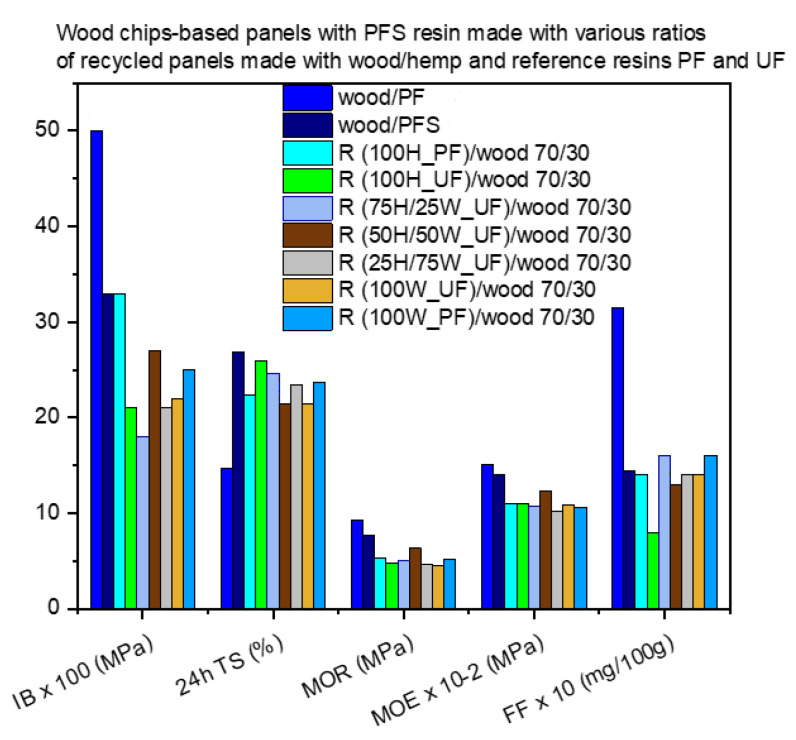
Performance of wood chip-based panels with recycled material received from panels made with reference resins and virgin wood chips/hemp shives at various ratios.

Figure 8 shows the properties of panels in which wood chips have been partially replaced by material obtained from the mechanical recycling of particleboards made with a mixture of wood chips and hemp shives in different proportions and the reference resins UF and PF. In this case, the end products were made with the PFS resin.

Figure 8 shows that the virgin wood chip panels have the best mechanical properties, whether they are made with PF or PFS resin. However, they have quite high formaldehyde content values and similar thickness swelling (24 h TS) behavior to the other panels, with the exception of the panel made with PF resin, which has significantly lower 24 h TS values than the other panels. This can be explained by the fact that the recycled material contains the resin from the first panel production, which has already penetrated and cured into the cells of the lignocellulosic biomass. These areas cannot form new bonds with the PFS resin during new panel production. As a result, the new board will have lower performance in all properties. These results are in line with the findings of other studies [18].

Among the panels produced with different levels of wood chip replacement by recycled material, the best overall results are observed when the recycled panels are made with 100% hemp shives and PF resin and when the biomass is 50/50 hemp shives/wood chips and a typical UF resin was used to produce the original panel. This can be explained by the fact that, as mentioned above, the recycled material does not have as many sites available for chemical reactions with the new resin in its second use cycle as the virgin biomass.

**Figure 9:**
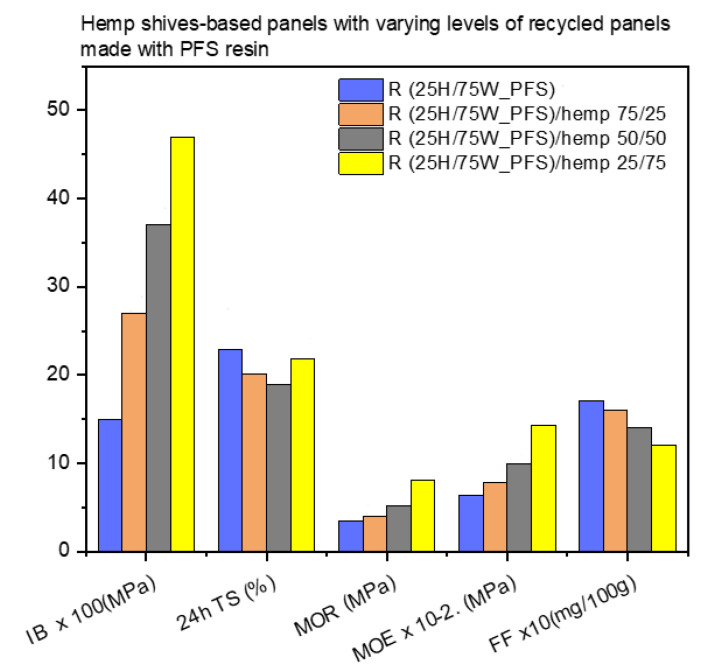
Performance of hemp shive-based panels with recycled material from panels made with PFS resin.

Regarding the panels produced by partially replacing virgin hemp shives with recycled panels (made with virgin hemp shives/wood chips in a 25/75 ratio and PFS resin), it can be seen in Figure 9 that the higher the virgin hemp shive content in the final panel, the better the mechanical properties (IB, MOR, MOE). No significant differences are observed in their thickness swelling behavior, while the formaldehyde content decreases as the level of virgin biomass in the panel increases. The results are consistent and expected because the recycled material used already contains a quantity of resin that contributes to the formaldehyde content of the final product while preventing good bonding of the materials with the new resin, which affects its mechanical properties.

**Figure 10:**
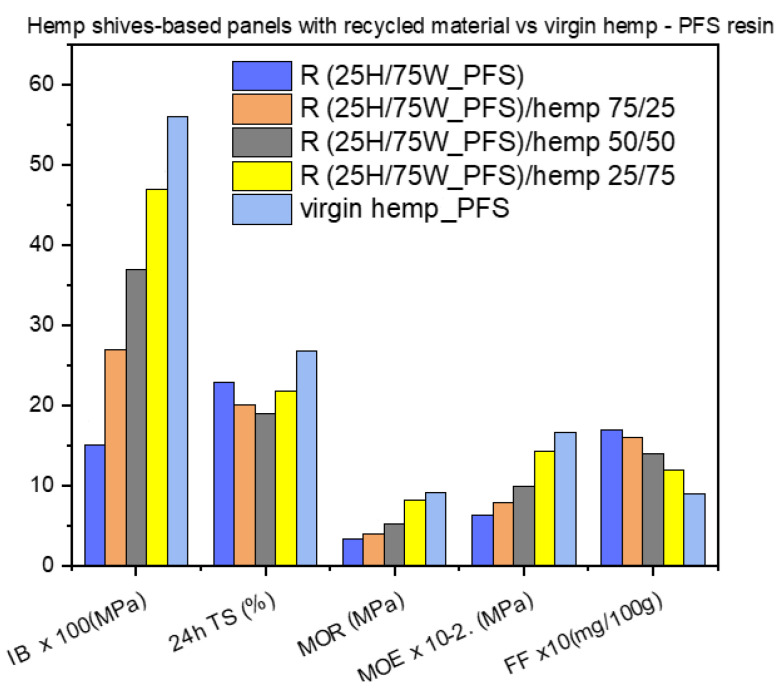
Performance of hemp shive-based panels with recycled material compared with panels made with virgin hemp shives.

In Figure 10, the above results with those of a panel made from 100% virgin hemp shives and PFS resin (Figure 7) are presented together for comparison. We can see that the same trend is followed and that the higher the content of virgin material, the higher the mechanical properties and the lower the formaldehyde content, while there is no significant effect on the thickness swelling (24 h TS).

## 4. Conclusions

The potential use of hemp shives in the production of particleboard is emphasized by the results of this study.

In particular, our research shows that not only can virgin hemp shives successfully replace wood chips in the production of lightweight particleboard, but that hemp shive-based panels can also be effectively recycled and reused in the production of new panels using up to 100% recycled material. However, the mechanical and physical properties of panels made from recycled material were slightly inferior to those made from virgin biomass. The study of the particleboards with FTIR confirmed the existence of resins and showed that recycling does not affect their chemical structure. In the case of particle boards with recycled material, there was a noticeable increase in their thermal stability. This indicates their enhanced resistance to thermal degradation, further enhancing their suitability for various applications.

Our research also investigated the use of soy flour in the synthesis of phenol–formaldehyde (PFS) type resins. The successful synthesis of the new soy-modified PF resin was confirmed by typical laboratory analysis and FTIR analysis, providing insight into its chemical structure. The adhesive performance of the resin was evaluated through its use in the production of lightweight wood chip- and hemp shive-based particle boards, and it was found that it can be successfully used as a binder for such boards made from either virgin or recycled biomass. Additionally, thermogravimetric analysis (TGA) revealed that the PFS resin exhibits similar thermal behavior to PF.

Recycling hemp shive panels helps diminish the demand for virgin materials, lowering the overall environmental impact associated with resource extraction and processing. Additionally, biobased resins, derived from renewable sources, contribute to a reduced carbon footprint compared to traditional petroleum-based alternatives.

This work highlights the potential for sustainability and resource efficiency in the particleboard industry.

## Figures and Tables

**Table 1:** Composition of Resins of Phenolic type.

Name of the Resin	Raw Materials	Ratio of Raw Materials in the Resin	Acronym of Resin
Phenol-Formaldehyde	Phenol	26.0%	PF
	Formaldehyde	45.5%	
	NaOH	14.8%	
	Water	13.6%	
Phenol-Formaldehyde-Soy flour	Phenol	19.5%	PFS
	Soy flour	6.5%	
	Formaldehyde	45.5%	
	NaOH	14.8%	
	Water	13.6%	

**Table 2:** Composition of UF resin.

Name of the Resin	Raw Materials	Ratio of Materials in the Resin	Acronym of Resin
Urea-Formaldehyde	Formaldehyde	40.3%	UF
	Urea-I	13.8%	
	Urea-II	13.8%	
	Water	32.1%	

**Table 3:** Panel types prepared and studied.

Virgin Biomass	Recycled Biomass	Ratio of Virgin/Recycled Biomass	Resin Used for the New Panels with Recycled Material	Abbreviation
Wood chips	-	100/0	PF, PFS, UF	wood-UF, wood-PF, wood-PFS25
Hemp shives	-	100/0	PF, PFS, UF	hemp-UF, hemp-PF, hemp-PFS25
Wood chips	Panels made from virgin wood chips/hemp shives at the ratios of: 0/100 and 100/0 bonded with PF and UF resins.	70/30	PFS	R(100H-PF)/wood R(100H-UF)/wood R(100W-PF)/wood R(100W-UF)/wood
Wood chips	Panels made from virgin wood chips/hemp shives at the ratios of: 25/75, 50/50, 75/25, bonded with UF resin	70/30	PFS	R(25H/75W-UF)/wood R(50H/50W-UF)/wood R(75H/25W-UF)/wood
Hemp shives	Panels made from virgin wood chips/hemp shives at the ratio of 25/75 bonded with PFS resin	0/100, 75/25, 50/50 and 25/75	PFS	R(25H/75W-PFS) R(25H/75W-PFS)/hemp 75/25 R(25H/75W-PFS)/hemp 50/50 R(25H/75W-PFS)/hemp 25/75

**Table 4:** Technical categories and specifications of particleboards according to their use (ΕΝ 312:2010).

Properties	Ιnternal Βond (IB) N/mm^2^	Bending Strength-(MOR) N/mm^2^	Modulus of Elasticity (MOE) N/mm^2^	Thickness Swelling (TS) %	
Test Method	EN319	EN310	EN310	EN317	
Category of Panel	Performance Requirements				Use
Ρ1	0.24	10	-	-	General-purpose boards for use in dry conditions
Ρ2	0.35	11	1600	-	Boards for interior fitments (including furniture) for use in dry conditions
Ρ3	0.45	14	1950	14	Non-load-bearing boards for use in humid conditions
Ρ4	0.35	15	2300	15	Load-bearing boards for use in dry conditions
Ρ5	0.45	16	2400	10	Load-bearing boards for use in humid conditions
Ρ6	0.50	18	3000	15	Heavy-duty load-bearing boards for use in dry conditions
Ρ7	0.70	20	3100	10	Heavy-duty load-bearing boards for use in humid conditions

**Table 5:** Classification of particleboards in terms of formaldehyde content according to the European standard (ΕΝ13986:2001).

Method for Formaldehyde Determination	Formaldehyde Content	Formaldehyde Class
Perforator method ΕΝ120/ΕΝ-ISO 12460-05:2015	≤8 mg/100 g oven dry board	Ε1
	>8 mg/100 g to ≤30 mg/100 g oven dry board	Ε2

**Table 6:** Properties of reference and experimental resins with various phenol replacement levels.

Resin	UF	PF	PFS
Solids, %	65.31	39.87	44.50
pH at 25 °C, [ ]	8.15	12.60	11.63
Viscosity, cP	250	343	515
Specific Gravity, [ ]	1.283	1.197	1.27
Water Tolerance, ml/ml	1/3.5	>1/9.0	>1/9.0
Gel Time, m	-	22	40
Gel Time, s	57	-	-
Free Formaldehyde, %	0.06	0.12	0.25
Alkali Content, %	-	8.52	7.65
Buffer Capacity, mL	11	-	-

## Data Availability

The data presented in this study are available in the manuscript (Particleboards with Recycled Material from Hemp-Based Panels).
